# Patient Engagement in Integrated Care: What Matters and Why?

**DOI:** 10.1111/hex.70146

**Published:** 2025-01-09

**Authors:** Marissa Bird, Nick Zonneveld, Francine Buchanan, Kerry Kuluski

**Affiliations:** ^1^ Trillium Health Partners, Institute for Better Health Mississauga Ontario Canada; ^2^ Institute of Health Policy, Management and Evaluation, University of Toronto Toronto Ontario Canada; ^3^ Department of Organization Studies School of Social and Behavioral Sciences, Tilburg University Tilburg The Netherlands; ^4^ Vilans–Center of Excellence for Care and Support Utrecht The Netherlands

1

This special issue endeavoured to solicit papers that were examining, advancing and ideally connecting the fields of patient, caregiver and community engagement with integrated care (defined as the connectivity between health and social care at the micro, meso or macro levels) [1]. Engagement and integrated care can be considered symbiotic: engaging with patients, caregivers with lived illness experience and community partners helps us to understand how we can better connect the dots between the often‐disparate health and social settings and resources, which is one of the goals of integrated care. Also, we cannot create systems that actually work for individuals and communities without engaging the public (i.e., users of the ‘system’) in shaping what they should look like. Ultimately, partnering with patients, caregivers and community members can advance and optimise the implementation and development of integrated care that is more meaningful for people who use health and social care.

In the existing body of literature on integrated care, it is not always clear the extent to which patients, caregivers and community are involved in its design, implementation and evaluation. Further, the level of engagement described in the literature is not always detailed and may be limited to one‐way communication, such as ad hoc consultations or surveys, which can be considered tokenistic if not fit for purpose. This Special Edition is unique in that it set out to find examples that brought the worlds of integrated care and engagement together, particularly deeper levels of engagement, like codesign and examples where patients, caregivers and communities were partners in research and decision making.

What surfaced in this collection of papers was a rich tapestry of codesign projects with a number of populations ranging from (and not limited to) young children with complex care needs [2], to adolescents at risk of suicide [3, 4], to pregnant and parenting women with substance use disorder [5], to older adults requiring palliative care [6]. Importantly, we saw examples of engagement with diverse communities and populations that are often left behind due to structural and social marginalisation [7, 8, 9] or high degrees of medical [2] or social complexity [5] providing important examples of people who have been historically excluded from research. In some studies, engagement was taken one step further by working with patient and caregiver partners as *members of the study team* or co‐researchers who participated in some or all stages of the research life cycle [6, 10, 11]. Whitmore et al trained people with type I diabetes as peer researchers, amplifying the impact of the research by recruiting a diverse group of people with diabetes for their study resulting in a rich codesign process and important insights related to optimising recruitment and integrating services for this population. In other cases, tools and interventions were codesigned to support better care experiences and coordination of services [11, 12] with the aim of implementing and sustaining these interventions over time.

Several important calls to action arose from this paper series, borne out of interviews, literature syntheses and the design of frameworks. These calls to action can guide us into the future as we seek to create more integrated systems of care where populations are engaged in its design, implementation, evaluation and evolution. These calls to action position patient partners as leaders in this endeavour [13], as essential members of care delivery teams [14] and shed light on the role of peers as essential connectors between underserved communities and care providers [15]. Furthermore, we were introduced to the Expanded Chronic Care Patient‐Professional Partnership Model (E2C3PM) which is designed to rebalance power between care providers, patients and their caregivers as they navigate the complexities of health systems [16].

Engagement methods such as codesign (featured prominently in this collection of papers) are effective in bringing together people with lived health care experience, people working at the front lines of health and social care and those with the power to implement change. By working in partnership, the detail and nuance required to embed findings into complex, disparately functioning health and social care systems can be unearthed. However, our current ‘project focused’ culture and limited bouts of funding creates a challenge in making fundamental, lasting, and adaptable changes needed to create a more interconnected system for people who use, deliver and manage health and social care. The importance of having continuous funding cycles, integrated care measures and academic and policy evaluation that take into consideration the time and challenges it takes for this work to happen is required.

## 2 Seeing the Forest for the Trees: Person‐Centred and Population‐Focused

As demonstrated by the articles included in this special issue and consistent with historical engagement efforts in healthcare, patients, caregivers, and community partners (PCC) have largely been engaged in healthcare research and operationalization efforts at the project level. Indeed, the ‘secret sauce’ of ‘good’ engagement has been touted as meaningfully engaging PCC in healthcare projects by incorporating their individual and unique experiences into all stages of healthcare research and programme design, implementation, and evaluation [17]. The democratisation of healthcare research and operationalization via PCC engagement has been demonstrated to improve research credibility and applicability to PCC [18], as well as improving health outcomes and experiences for PCC by tailoring programs to suit their needs [19].

However, reliance on solely project‐based PCC engagement may be problematic for integrated care. The central defining features of integrated care include continuity and coordination of health and social care service [20]. Continuity and coordination are achieved through the pursuit of an organised set of activities aimed at integrating health and social care services for communities and populations. Goodwin [1] argues that integration of care is a way to bring together health and social service assets at the community level such that the focus of integrated care extends beyond service models for individual users that fit a specific clinical profile to promoting health and wellbeing for populations.

## 3 Call to Action

Bearing this in mind, engagement in integrated care must extend beyond a project‐based and time‐limited nature to one that longitudinally and meaningfully engages with entire communities. Integrated care practitioners must *both* seize the opportunity of partnership as a chance to engage with the person in front of us, in all their uniqueness, *and* in tandem hold the needs of the population in our mind's eye. In other words, PCC engagement in integrated care must both acknowledge and celebrate the lived experience brought by each individual, while also ‘zooming out’ to engage with the needs of the population.

Engaging with the needs of the population will enable us to move beyond a focus on acute care needs to centre care on community needs, a focus on prevention, and acknowledging and addressing multiple determinants of health through partnerships within communities [21]. In addition, PCC engagement focused on a population health approach may help to address longstanding equity issues in traditional PCC engagement. When we rely on the voices of a select few to speak for the collective many, segments of the population are by necessity left behind. Many times, justification for the selection of PCC partners is not documented by researchers, but criteria often cited are interest, convenience, and availability. Often, the few partners selected for engagement are not representative of the diversity of the population, potentially exacerbating systematic oppression and exclusion of groups whose voices are not captured in health research and operationalization engagement efforts. Finding solutions to the person‐centred and population‐focused paradox requires a focus on extending engagement beyond time‐limited projects, as well as harnessing PCC input on a broader scale than single voices. Potential paths forward for this type of dual‐focused engagement exist, such as ecological approaches that account for the interaction between individuals and the wider health ecosystem [22].

We encourage those working at the intersection of integrated care and patient engagement to consider both the person and the population in their engagement efforts.

## Author Contributions

MB and KK drafted the initial version of the manuscript, NZ and FB contributed to critically revising the manuscript. All authors have approved the final version.

## Conflicts of Interest

The authors declare no conflicts of interest.

## Data Availability

The authors have nothing to report.
